# Progress toward Understanding Protein S-acylation: Prospective in Plants

**DOI:** 10.3389/fpls.2017.00346

**Published:** 2017-03-24

**Authors:** Yaxiao Li, Baoxiu Qi

**Affiliations:** Department of Biology and Biochemistry, University of BathBath, UK

**Keywords:** lipid modification, S-acylation, PATs, substrate recognition and specificity, yeast, mammalian, plants

## Abstract

S-acylation, also known as S-palmitoylation or palmitoylation, is a reversible post-translational lipid modification in which long chain fatty acid, usually the 16-carbon palmitate, covalently attaches to a cysteine residue(s) throughout the protein via a thioester bond. It is involved in an array of important biological processes during growth and development, reproduction and stress responses in plant. S-acylation is a ubiquitous mechanism in eukaryotes catalyzed by a family of enzymes called Protein S-Acyl Transferases (PATs). Since the discovery of the first PAT in yeast in 2002 research in S-acylation has accelerated in the mammalian system and followed by in plant. However, it is still a difficult field to study due to the large number of PATs and even larger number of putative S-acylated substrate proteins they modify in each genome. This is coupled with drawbacks in the techniques used to study S-acylation, leading to the slower progress in this field compared to protein phosphorylation, for example. In this review we will summarize the discoveries made so far based on knowledge learnt from the characterization of protein S-acyltransferases and the S-acylated proteins, the interaction mechanisms between PAT and its specific substrate protein(s) in yeast and mammals. Research in protein S-acylation and PATs in plants will also be covered although this area is currently less well studied in yeast and mammalian systems.

## Introduction

**Figure 1:**
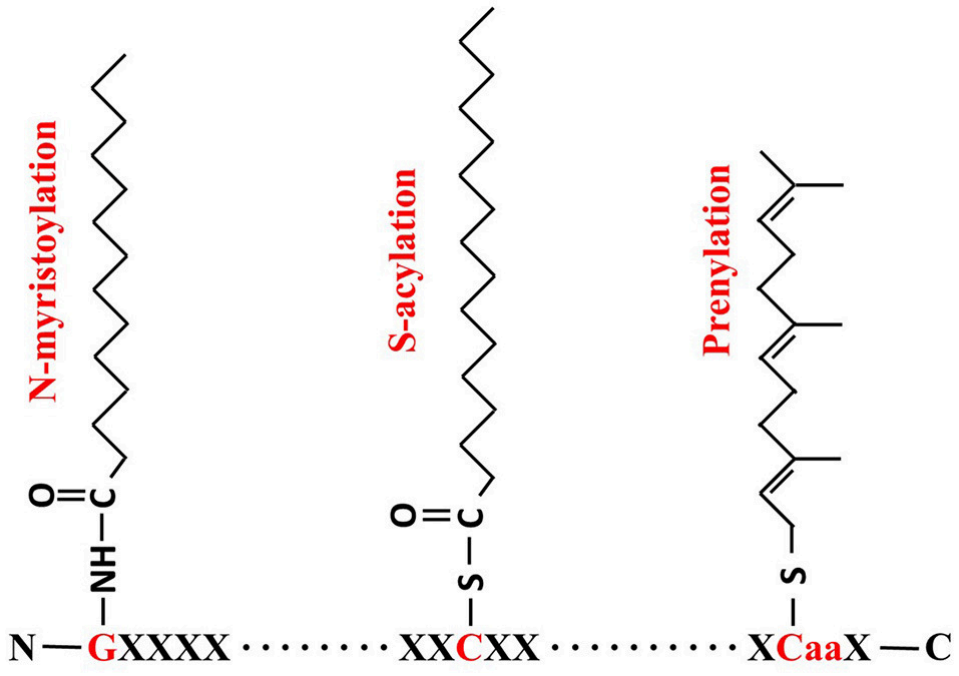
**Formulae of N-myristoylation, S-acylation and prenylation**. For N- myristoylation, a 14-carbon myristoyl group is covalently attached by an amide bond to the alpha-amino group of an N-terminal glycine (G, in red); S-acylation is the attachment of a 16-carbon palmitate to cysteine residue (C, in red) via thioester bond; and Prenylation makes a 15-carbon farnesyl link to the CaaX cysteine residue in C-termini.

Lipid modification is a common mechanism in organisms, in which a fatty acid attaches to specific amino acid residues, leading to increased hydrophobicity of proteins which aids their anchoring to membranes or specific lipid rafts (Levental et al., 2010). The three most commonly known lipid modifications are N-myristoylation, prenylation and S-acylation (Figure 1). N-myristoylation is an irreversible, co-translational protein modification in which 14-carbon myristoyl group is covalently attached to N-terminal glycine residue via an amide bond (Martin et al., 2011). Prenylation is a post-translational lipid modification which involves the transfer of either a 15-carbon farnesyl or a 20-carbon geranyl-geranyl moiety to CaaX C-terminal cysteine of the target protein. S-acylation, more commonly known as S-palmitoylation, is a post-translational lipid modification in which a long chain fatty acid, usually the 16-carbon palmitate, covalently attaches to the specific cysteine residue(s) throughout the protein via a thioester bond (Resh, 2006; Greaves and Chamberlain, 2011).

It is noteworthy that three types of protein palmitoylation are found so far, including S-palmitoylation, N-palmitoylation and O-palmitoylation. While S-palmitoylation can occur at any Cys residues along the protein sequence in which the palmitate is reversibly attached via thioester bond as shown in Figure 1, N-palmitoylation is a stable lipid modification at the N-terminal residue (very often Cys) through amide linkage. A small group of secreted proteins have been identified as N-palmitoylated proteins, including the epidermal growth factor (EGF) like ligand “Spitz” and Hedgehog family members in Drosophila and mammals (Pepinsky et al., 1998; Chamoun et al., 2001; Miura et al., 2006; Buglino and Resh, 2012). Since N-palmitoylation can be easily converted by S-palmitoyl migration, it is still not very clear whether N-palmitoylation is an independent enzyme-catalyzed reaction or just from S- to N-palmitoyl transfer (Ji et al., 2016). Less frequently, palmitoyl group can also be linked to a serine residue through ester bond via the so-called O-palmitoyltion. The identified O-palmitoylated targets so far include Wnt/Wg proteins and the peptide hormone preghrelin (Takada et al., 2006; Yang et al., 2008). Although, palmitate is thought to be the most common fatty acid found to be attached to S-palmitoylated proteins recent studies proved that other acyl groups such as stearate (C18:0) or oleate (C18:1) are also accepted in S-palmitoylation. Therefore, S-acylation is a more representative term than palmitoylation (Jones et al., 1997; Sorek et al., 2007; Hurst and Hemsley, 2015). In contrast to other lipid modification, such as myristoylation, prenylation, N-palmitoylation or O-palmitoylation, S-acylation is a unique posttranslational modification in that it is usually reversible (Fukata and Fukata, 2010). As such it is important for cellular protein sorting, vesicle trafficking, activation state control, protein stability, membrane microdomain partitioning of protein and protein complex assembly (Greaves and Chamberlain, 2007; Baekkeskov and Kanaani, 2009; Charollais and Van Der Goot, 2009; Hemsley, 2009; Hemsley et al., 2013).

Some other lipid modifications, such as Glycosylphosphatidylinositol (GPI) and glycosylinositolphosphorylceramide (GIPC) anchors can link the whole glycolipids to the protein instead of the simple fatty acid or polyisoprene group (Hemsley, 2015). GPI and GIPC anchors modify proteins at the lumen side instead of in the cytosol as do the other three lipid modifications (Ganesan and Levental, 2015). Lipid modifications which are only found in specific proteins, such as cholesterol addition at the C-terminal glycine of proteins have also been reported (Buglino and Resh, 2012). All these lipid modifications are widely present in mammals and plants (except for N- or O- palmitoylation which is only found in mammals so far) and all play important roles during growth and development through the modification of an array of proteins.

Although all lipid modifications can facilitate the attachment of proteins to membranes, modification with palmitoyl groups provide more affinity, about 10 times stronger than myristoyl groups and 100 times than farnesyl groups (Silvius and L'heureux, 1994; Hemsley, 2009).

## S-acylation

S-acylation can occur both on soluble and transmembrane proteins (Roth et al., 2006; Blaskovic et al., 2013). S-acylation of soluble proteins allows their association with membranes, trafficking, regulation and signaling (Roth et al., 2006; Blaskovic et al., 2013). For example, a constitutive de/re-acylation of H- and N- small Rat sarcoma (Ras) drives their subcellular localization from plasma membrane (PM) to Golgi which initiates RAS activation (Rocks et al., 2005). Although, the direct mechanism of S-acylation on transmembrane proteins is not very clear it is thought that it plays multiple roles in altering signaling capacity (Merrick et al., 2011), reducing activity (Huang et al., 2010), trafficking modification (Abrami et al., 2008; Flannery et al., 2010) and changing stability of these proteins (Abrami et al., 2006; Maeda et al., 2010; Blaskovic et al., 2013). For example, S-acylation of transmembrane proteins, such as death receptor 4 (Oh et al., 2012), β-secretase BACE1 (Motoki et al., 2012), cannabinoid receptor (Oddi et al., 2012) and influenza virus M2 protein (Thaa et al., 2011), can promote their association with membrane lipid rafts. However, for some peripheral membrane proteins such as transferrin receptor and caveolin, their palmitoylation sites are localized to non-raft domains, therefore palmitoylation is not necessary for their raft localization (Alvarez et al., 1990; Dietzen et al., 1995; Charollais and Van Der Goot, 2009). In the case of the tumor endothelial marker 8 (TEM8) palmitoylation was actually found to negatively regulate its raft association (Abrami et al., 2006).

### S-acylation in yeast

A proteomic method using the acyl-biotinyl exchange (ABE) chemistry combining with the traditional [^3^H] palmitate *in vivo* labeling protocol identified 48 S-acylated proteins that span a wide range of cellular functions in *Saccharomyces cerevisiae* (Roth et al., 2006). These include a large number of SNAREs (soluble N-ethylmaleimide-sensitive fusion protein-attachment protein receptor) that are involved in vesicle fusion. Redundant SNAREs, such as plasma membrane (PM) localized synaptobrevin homologs Snc1 and Snc2, were first identified to be S-acylated proteins in 1995 (Couve et al., 1995), and subsequently confirmed independently (Valdez-Taubas and Pelham, 2005; Roth et al., 2006). Ykt6 is another commonly known S-acylated SNARE. It requires both C-terminal prenylation and palmitoylation to target to the membrane, which is different from all other single transmembrane domain (TMD) containing SNAREs (Fukasawa et al., 2004). Tlg1 lacking S-acylation undergoes ubiquitination, implying S-acylation can protect proteins from degradation (Valdez-Taubas and Pelham, 2005). Other SNAREs that have been confirmed to be S-acylated are Sso1, Sso2, Vam3, Tlg2, and Syn8 (Valdez-Taubas and Pelham, 2005; Roth et al., 2006). S-acylation is also very common in many important signaling proteins, such as the heterotrimeric G protein alpha and gamma subunits Gpa1 (Song and Dohlman, 1996; Song et al., 1996), Gpa2 (Harashima and Heitman, 2005), and Gγ (Ste18, Hirschman and Jenness, 1999); small monomeric G proteins (GTPases) such as Rho1, Rho2 (Roth et al., 2006), Rho3 (Zhang et al., 2013), Ras1 and Ras2 (Deschenes et al., 1990; Mitchell et al., 1994; Bartels et al., 1999). A recent study shows that the pathogenesis, morphogenesis and sexual differentiation of an encapsulated yeast *Cryptococcus neoformans* is achieved through the important roles that S-acylation plays in modulating the localization of Ras1 (Nichols et al., 2015). Interestingly, all of these signaling proteins acquire prenylation or myristoylation before S-acylation occurs (Roth et al., 2006).

In addition, many amino acid permeases (AAP) were proved to be S-acylated (Roth et al., 2006). For example, the yeast type I casein kinases, Yck1, Yck2, and Yck3, which play important roles in cellular morphology, bud emergence and endocytosis of mating pheromone receptor, are membrane localized via S-acylation for function (Roth et al., 2006, 2011). ENV7 (late endosome and vacuole interface) encodes a protein kinase that plays important roles in vacuole morphology, and its proper membrane localization and function relies on S-acylation of the N-terminal triple cysteines motif (C^13^C^14^C^15^) (Manandhar et al., 2013, 2014; Cocca, 2014). S-acylation of telomere-binding protein Rif1 anchored it to the inner nuclear membrane, which influences its role in heterochromatin dynamics (Park et al., 2011). Mutagenesis of cysteine in different positions of Arsenite permease Acr3p can cause its completely or partially dysfunction as a low affinity As(III)/H^+^ and Sb(III)/H^+^ antiporter, and Cys90 which localizes in the cytosolic loop but in close proximity to transmembrane regions has the high possibility to be S-acylated (Maciaszczyk-Dziubinska et al., 2014). It was also reported that S-acylation is necessary for the export of chitin synthase Chs3 from ER (Lam et al., 2006). The information described in this section is summarized in Table 1.

**Table 1:** **Individually confirmed S-acylated proteins in yeast**.

**Groups**	**Specific proteins**	**References**
SNAREs	Snc1/2, Ykt6,Tlg1/2, Sso1/2,Vam3, Syn8	Couve et al., 1995; Fukasawa et al., 2004; Valdez-Taubas and Pelham, 2005; Roth et al., 2006
G proteins	Gpa1/2, Ste18, Rho1/2/3, Ras1/2	Deschenes et al., 1990; Mitchell et al., 1994; Song and Dohlman, 1996; Song et al., 1996; Bartels et al., 1999; Hirschman and Jenness, 1999; Harashima and Heitman, 2005; Roth et al., 2006; Zhang et al., 2013; Nichols et al., 2015
AAPs	Tat1/2, Gnp1, Sam3, Hip1, Bap2, Agp1, Gap1	Roth et al., 2006
Protein kinases	Yck1/2/3, Env7	Roth et al., 2006, 2011; Cocca, 2014
Other proteins	Rif1, Acr3p, Chs3	Lam et al., 2006; Park et al., 2011; Maciaszczyk-Dziubinska et al., 2014

### S-acylation in mammals

Following study of S-acylation in yeast research that has extended to mammalian systems considerable knowledge has been gained in recent years, revealing the involvement of protein S-acylation in the regulation of growth, development, and cancer and disease status. For example, a global rat neural palmitoyl-proteome characterized almost 300 S-acylated proteins, again with the ABE method adapted from the yeast study (Kang et al., 2008). Similarly 331 S-acylated proteins were identified from human prostate cancer cells (Yang et al., 2010), 57 from human B lymphoid cells (Ivaldi et al., 2012) and 150 from endothelial cells (Marin et al., 2012). By bio-orthogonal labeling of S-acylated proteins with 17-octadecynoic acid (ODYA) about 125 and over 400 S-acylated proteins were identified from human Jurkat T-cells and mouse T-cell hybridoma cells, respectively (Martin and Cravatt, 2009; Martin et al., 2012).

It is worth noting that proteins that have been proved to be S-acylated in yeast, their homologous proteins in mammals tend to be also S-acylated. For instance, many human SNAREs were proved to be also S-acylated (Greaves et al., 2010), S-acylation of α subunits of G proteins is necessary for their membrane localization and function (Wedegaertner et al., 1993; Grassie et al., 1994; Ponimaskin et al., 1998, 2000). However, G-protein γ subunits have not been reported to be S-acylated in mammals. Many G-protein-coupled receptors (GPCRs) (Blaskovic et al., 2013) and Ras GTPase (Rocks et al., 2005) are also S-acylated. Mitochondrial targeting of a microphage protein phospholipid scramblase 3 (Plscr3) is dependent on its S-acylation (Merrick et al., 2011).

Some S-acylated proteins in mammals can also make themselves avoid degradation by attaching a palmitate molecule. For instance, LRP6 (lipoprotein-receptor-related protein 6) is S-acylated and the removal of acyl group leads to destabilization or ubiquitination (Abrami et al., 2008). Similarly, the palmitoylation of TEM8 (Abrami et al., 2006), CCR5 (chemokine and HIV receptor) (Percherancier et al., 2001) and Rhodopsins (Maeda et al., 2010) prevents the degradation of these proteins. It was also reported that for some other proteins, their degradation depends on the S-acylation. For example, a cancer-promoting protein CDCP1 (CUB domain-containing protein 1) is degraded upon S-acylation, leading to a decrease of ovarian cancer cell migration (Adams et al., 2015). Therefore, it seems that S-acylation can play opposite roles in protein degradation.

Many signaling proteins involved in keeping T-cell homeostasis are S-acylated, such as T-cell co-receptors CD4 and CD8, tyrosine kinases Lck and Fyn, and adaptor proteins LAT (linker for activation of T cells) and Cbp/PAG (Bijlmakers, 2009; Hundt et al., 2009; Akimzhanov and Boehning, 2015). S-acylation of Lck at both Cys3 and Cys5, which are redundant for the function of Lck, is essential for propagating T-cell receptor signaling and releasing apoptotic calcium (Akimzhanov and Boehning, 2015). Similarly, LAT is also a dual (Cys26 and Cys29) S-acylated protein which is required for T cell development and activation. However, S-acylation of Cys26 alone is enough for its PM localization and proper function (Hundt et al., 2009).

S-acylation of synaptic proteins is important for synaptic plasticity, and the key S-acylated synaptic proteins include postsynaptic density protein PSD-95, δ-catenin, gephyrin, A-kinase anchoring protein AKAP79 and 150, the small GTPase Cdc42. Lack of S-acylation of these proteins lead to impaired performance on learning and memory tasks (Brigidi et al., 2015). Huntington's disease is a neurodegenerative disorder caused by mutation in the gene encoding the S-acylated Huntingtin (HTT) (Butland et al., 2014). Defects in S-acylation can also cause mental problems such as schizophrenia and X-linked mental retardation (XLMR), however, the specific S-acylated target proteins involved in this process have not been isolated (Mukai et al., 2004; Raymond et al., 2007). Alzheimer's disease (AD) is a neurodegenerative dementia which accounts for 60–70% of cases of dementia. Many studies have demonstrated that S-acylation plays very important roles in the pathogenesis of AD, and the related S-acylated proteins include β- and γ-secretase enzymes, and the major APP (amyloid precursor protein) cleaving enzyme BACE1, which are S-acylated at four sites (Benjannet et al., 2001; Hornemann, 2015).

Autophagic protein microtubule-associated protein 1 light chain-3B (LC3B) is a positive regulator of chronic obstructive pulmonary diseases such as emphysema. LC3B is associated with the extrinsic apoptotic factor Fas, and their interaction is mediated by caveolin-1 (Cav-1). Interestingly, both Fas and Cav-1 are S-acylated proteins (Chen et al., 2010). S-acylation of the bone developmental regulator membrane type1-metalloprotease (MT1-MMP) is a key modulator of bone homeostasis (Song et al., 2014). Goltz syndrome, caused by loss of function of the S-acylated protein Porcupine (Galli et al., 2007; Hornemann, 2015), is an X-linked dominant form of ectodermal dysplasia, which is primarily characterized by skin manifestations as atrophic and hypoplastic areas and results in osseous defects and dental anomalies later (Wang et al., 2007).

An increasing number of reports indicate that S-acylation is involved in cancer. For instance, Ras is a negative regulator of cell proliferation, and S-acylation of Ras maintains its steady state plasma membrane localization which is essential for transduction of extracellular proliferative signals (Rocks et al., 2005; Schmick et al., 2015). S-acylation of the neurotensin receptor 1 (NTSR-1), a key mediator in breast, pancreas, prostate, colon and lung cancers, is essential for its localization and efficient signaling (Heakal et al., 2011). The induction of apoptosis is an efficient way to stop tumor development, many proteins involved in apoptosis are S-acylated including FasL (Fas Ligand; Guardiola-Serrano et al., 2010), FasR (Fas receptor; Chakrabandhu et al., 2007), DR4 (a receptor of the tumor necrosis factor-related apoptosis-inducing ligand; Rossin et al., 2009), DCC (deleted in colorectal cancer; Furne et al., 2006), UNC5H (Maisse et al., 2008) and BAX (BCL-2-associated X) (Fröhlich et al., 2014). The spread of cancer cells from their original site to other parts of the body is through metastasis. It was reported that S-acylation of metastasis-associated proteins KAT1/CD82, CD9, and CD151 is essential for their function of suppressing metastasis or inhibiting tumor cell adhesion and migration (Zhou et al., 2004; Hemler, 2014; Termini et al., 2014). Integrin β4 (ITGβ4) can interact with growth factor receptors and enhance invasive potential of cancer cells (Soung and Chung, 2011). This is helped by the S-acylation of ITGβ4 which is required for its lipid raft localization in the membrane and signaling activity. The level of ITGβ4 S-acylation is correlated with the invasive potential of breast cancer cells (Coleman et al., 2015). Another S-acylated protein related to breast cancer is CD44 which negatively regulates cell migration (Xie et al., 2009). Endothelial nitric oxide synthase (eNOS), which localizes through S-acylation to the Golgi complex and PM cholesterol-rich microdomains, promotes angiogenesis and tumorigenesis (Fernández-Hernando et al., 2006; Wei et al., 2011). Table 2 lists the identified S-acylated proteins in mammals described in this section.

**Table 2:** **S-acylated proteins individually verified in mammalian cells**.

**Groups**	**Examples**	**References**
SNAREs	SNAP23, SNAP25, SNAP25b	Greaves and Chamberlain, 2010; Greaves et al., 2010
G Proteins	Go1α, Gα_12_, Gα_13_, GPCRs, GTPase	Wedegaertner et al., 1993; Grassie et al., 1994; Ponimaskin et al., 1998, 2000; Rocks et al., 2005
T-cell specific proteins	CD4/8, Lck, Fyn, LAT, Cbp/PAG	Bijlmakers, 2009; Hundt et al., 2009; Akimzhanov and Boehning, 2015
B-cell specific proteins	CD20/23	Ivaldi et al., 2012
Synaptic proteins	PSD-95, δ-catenin, gephyrin, AKAP79/150, Cdc42, HTT, β- and γ- secretases, BACE1	Benjannet et al., 2001; Butland et al., 2014; Brigidi et al., 2015; Hornemann, 2015
Cancer related proteins	CDCP1, Ras, NTSR-1, FasL, FasR, DR4, DCC, UNC5H, BAX, CD82/9/151/44, ITGβ4, Enos	Zhou et al., 2004; Fernández-Hernando et al., 2006; Furne et al., 2006; Chakrabandhu et al., 2007; Maisse et al., 2008; Rossin et al., 2009; Xie et al., 2009; Guardiola-Serrano et al., 2010; Heakal et al., 2011; Soung and Chung, 2011; Wei et al., 2011; Fröhlich et al., 2014; Hemler, 2014; Termini et al., 2014; Adams et al., 2015; Coleman et al., 2015; Schmick et al., 2015
Other Proteins	Plscr3, LRP6, Fas, Cav-1, MT1-MMP; Porcupine, TEM8, CCR5	Abrami et al., 2006, 2008; Chen et al., 2010; Galli et al., 2007; Merrick et al., 2011; Percherancier et al., 2001; Song et al., 2014

The above studies clearly demonstrate that S-acylation is involved in a wide range of human diseases including mal-development, infectious diseases, autoimmune diseases, neuropsychiatric disorders, dermatosis, osteoporosis, and cancer (Ivaldi et al., 2012; Chavda et al., 2014; Hornemann, 2015; Yeste-Velasco et al., 2015). Understanding S-acylation will provide invaluable information to the insight of disease processes which in turn will aid the development of drugs to control and target these various diseases. Therefore, the relationship between protein S-acylation and disease in human becomes a hot research topic in the medical field in recent years.

### S-acylation in plants

Our understanding of plant S-acylation is rudimentary and the limited knowledge comes mainly from targeted studies on the functional characterization of individual proteins that happen to be S-acylated, including, mainly heterotrimeric G protein and some small monomeric G-proteins. For instance, the α subunit GPA1 and γ subunit AGG2 of plant heterotrimeric G protein are S-acylated. GPA1 has dual lipid modification with a myristoylation site at the G2 position and an adjacent S-acylation site at the C5 position, ensuring its localization to the PM (Adjobo-Hermans et al., 2006). Apart from promoting PM localization, S-acylation of GPA1 may also stabilize the newly formed heterotrimer. AGG2 is S-acylated at Golgi before delivered to the PM, and its membrane localization is dependent on its prenylation and S-acylation (Zeng et al., 2007). Therefore, S-acylation may act as a membrane targeting signal and restricts AGG2 shuttle in and out of PM (Zeng et al., 2007; Hemsley, 2009). Some small GTPases are also known to be S-acylated. For instance, S-acylation of AtROP6 is responsible for its activation and inactivation cycles (Sorek et al., 2007). AtROP9 and AtROP10, which are involved in ABA signaling, contain 3 and 2 S-acylation sites respectively (Lavy et al., 2002; Zheng et al., 2002; Hemsley, 2009). For AtRABF1 (ARA6), both S-acylation and myristoylation are essential for its prevacuolar compartment localization (Ueda et al., 2001).

Proteins involved in Ca^2+^ signaling such as calcineurin B-Like proteins AtCBL1, AtCBL2, AtCBL3, and AtCBL6 in Arabidopsis (Batistic et al., 2008, 2012); calcium dependent protein kinases OsCPK2 in rice (Martin and Busconi, 2000); LeCPK1 in tomato (Leclercq et al., 2005); MtCPK3 in *Medicago truncatula* (Gargantini et al., 2006) and StCDPK1 in *Solanum tuberosum* (Raíces et al., 2003) were reported to be S-acylated. AtCBL1 is a dually lipid modified protein, in which myristoylation targets it to the endoplasmic reticulum (ER), but the trafficking from ER to PM and subsequent PM anchoring depends on S-acylation (Batistic et al., 2008).

Other S-acylated proteins are the pathogenesis related proteins such as RPM1 interacting protein 4 (RIN4) and leucine-rich repeat receptor like kinase (FLS2) (Kim et al., 2005; Hemsley et al., 2013; Running, 2014; Boyle et al., 2016); NDR1/HIN1-like (NHL) stress response proteins (Hemsley et al., 2013; Hurst and Hemsley, 2015); POLTERGEIST (POL) and POLTERGEIST LIKE 1 (PLL1) (their PM localization is dependent on both myristoylation and S-acylation at their N-termini) (Gagne and Clark, 2010); the Lost In Pollen tube guidance 1 (LIP1) and 2 (LIP2), mutations of their S-acylation sites abolished PM localization. Although, individual knockout mutant of LIP1 and LIP2 did not have any defects the double mutant can cause sterility due to loss of pollen tube guidance (Liu et al., 2013). S-acylation of remorin proteins, a group of well-known plasma membrane marker proteins, contribute to their subcellular localization (Konrad et al., 2014). Very recently, Kumar and his coworkers confirmed that a number of the catalytic subunits of cellulose synthase complex (CSC) in Arabidopsis are S-acylated. These include the cellulose synthase A 1 (CESA1), CESA4, CESA6, CESA7, and CESA8 where up to 6 S-acylation sites were in each of these proteins (Kumar et al., 2016). SGN1, a receptor-like cytoplasmic kinase (RLCK), localizes in a strictly polar fashion to the endodermal outer plasma membrane, and this is dependent on the S-acylation of N-termini (Alassimone et al., 2016; See summary in Table 3).

**Table 3:** **S-acylated proteins individually identified in plants**.

**Groups**	**Examples**	**References**
SNAREs	AtSYP71, AtSYP122, AtNPSN11	Hemsley et al., 2013
G-proteins	AtGPA1, AtAGG2, AtROP6/9/10, AtRABF1	Ueda et al., 2001; Lavy et al., 2002; Zheng et al., 2002; Adjobo-Hermans et al., 2006; Sorek et al., 2007; Zeng et al., 2007
Proteins in Ca^2+^ signaling	AtCBL1/2/3/6, OsCPK2, LeCPK1, MtCPK3, StCDPK1	Martin and Busconi, 2000; Raíces et al., 2003; Leclercq et al., 2005; Gargantini et al., 2006; Batistic et al., 2008, 2012
Cellulose Synthase complex	AtCESA1, AtCESA4, AtCESA6, AtCESA7, AtCESA8	Kumar et al., 2016
Others	RIN4, FLS2, POL, PLL1, LIP1, LIP2, remorins, SGN1	Kim et al., 2005; Gagne and Clark, 2010; Hemsley et al., 2013; Running, 2014; Liu et al., 2013; Konrad et al., 2014; Alassimone et al., 2016

On a proteomic level Hemsley and coworkers identified about 600 putative S-acylated proteins from Arabidopsis using a biotin switch isobaric tagging for relative and absolute quantification (Hemsley et al., 2013). These proteins are involved in many processes across plant growth, development and stress responses, including the mitogen-activated protein kinases (MAPKs), leucine-rich repeat receptor-like kinases (LRR-RLKs) and RLK superfamily members, integral membrane transporters, ATPases, SNAREs and others. Similarly, about 450 S-acylated proteins were identified from Poplar cell suspension very recently. Except for the commonly known intracellular trafficking related proteins such as protein kinases, SNAREs, band 7 family proteins and tetraspanins, some cell wall related proteins were also found to be S-acylated (Srivastava et al., 2016). These results greatly expand the range of functions of protein S-acylation involves in plants, demonstrating the important roles of protein S-acylation in plant growth, development and stress signaling.

### S-acylation in other organisms

S-acylated proteins were also identified from other organisms. For example, more than 400 putative S-acylated proteins were isolated from the most severe human malaria causing parasite *Plasmodium falciparum*, involved in almost all the stages of its life cycle (Hodson et al., 2015). A number of S-acylated proteins are localized in the inner membrane complex (IMC). IMC is a membranous two layered structure located underneath the plasma membrane, which IMC plays central roles in host cell invasion and cytokinesis in *P. falciparum* (Cavalier-Smith, 1993; Wetzel et al., 2015). In another parasite *Toxoplasma gondii*, S-acylated proteins were also proved to be involved in many physiological processes including motility, invasion and division (Beck et al., 2010; Frénal et al., 2010, 2014). In *Aspergillus fumigatus*, one of the most common species that cause the invasive aspergillosis in individuals with an immunodeficiency, Ras pathway signaling is its critical virulence determinant and the properly localized and activated Ras is dependent on a series of posttranslational lipid modification including S-acylation (Al Abdallah and Fortwendel, 2015). Study also showed that S-acylation is essential for spermatogenesis of *Caenorhabditis elegans* (Gleason et al., 2006).

S-acylation not only occurs on proteins synthesized in eukaryotic cells but also for proteins secreted by prokaryotic bacteria and viruses and subsequently S-acylated by their eukaryotic hosts. For example, *Legionella* and other bacterial pathogens can secrete effectors that mimic the substrates of host lipid transferases, which can help them target the proper host membranes after S-acylation and other lipid modifications (Ivanov and Roy, 2013). A group of cysteine protease type III effectors secreted by the plant pathogen *Pseudomonas syringae*, rely on their S-acylation by the host cells to be targeted to plasma membrane and activated (Dowen et al., 2009). S-acylation can also take place on viral proteins and in fact the first reported S-acylated protein was a glycoprotein from *Vesicular stomatitis* virus (Schmidt and Schlesinger, 1979; Hurst and Hemsley, 2015). Another viral S-acylated protein is the hemagglutinin of Influenza virus. S-acylation of its all three cysteine residues by the host cell S-acylation machinery is essential for the replication and infection of the virus (Zurcher et al., 1994; Wagner et al., 2005; Brett et al., 2014).

## Protein S-Acyl transferases (PATs)

While spontaneous palmitoylation does occur on some proteins in the cells (i.e., Bizzozero et al., 2001; Kümmel et al., 2006; Kostiuk et al., 2008) it is generally accepted that S-acylation is an enzymatic process catalyzed by a family of proteins, the Protein S-Acyl Transferases (PATs for short). This is because research on PATs was much delayed compared to that on the S-acylated proteins. The first PAT, Akr1 was identified from *S. cerevisiae* in 2002 which is 20 years later than S-acylation of protein first reported (Schmidt and Schlesinger, 1979; Bartels et al., 1999; Roth et al., 2002). Since then, the significance of this enzyme family has been gradually recognized by studies carried out by an increasing number of researchers in this field, leading to the great enrichment of our knowledge of PATs, particularly in yeast and mammals.

### The structure and functional domains of PATs

Compared to the numbers of enzymes that catalyze the N-myristoylation or prenylation, there are much more DHHC-containing PATs existing in eukaryotes. In contrast to the cytoplasmic catalyzing enzymes for S-prenylation and N-myristoylation, PATs are transmembrane proteins with 4-6 TMDs and cytosolic N- and C- terminii (Hemsley et al., 2013). Most importantly, PATs also have a highly conserved catalytic Asp-His-His-Cys Cysteine Rich Domain (DHHC-CRD) of ~50 amino acids (Roth et al., 2002). This domain was proposed as Cx2Cx9HCx2Cx4DHHCx5Cx4Nx3F (Mitchell et al., 2006), usually residing on the cytoplasmic face of membranes between transmembrane domains (TMD) 2 and 3 of PATs (Gottlieb et al., 2015). It was reported that mutation of cysteine in DHHC domain inhibits both acyl intermediate formation and acyl chain transfer activity of PATs (Mitchell et al., 2006; Gottlieb et al., 2015). Indeed, when cysteine residue in the DHHC motif of AtPAT24, AtPAT10 and AtPAT14 of Arabidopsis was mutated to alanine or serine, all 3 AtPATs lost their PAT activities (Hemsley et al., 2005; Qi et al., 2013; Li et al., 2016). The DHHC-CRD domain in Swf1 cannot be replaced by those from Pfa3, Pfa4 or Erf2, and similar results were also found for Pfa3, the DHHC-CRD of which cannot be replaced by that of Swf1 or Erf2. The irreplaceability of DHHC-CRD demonstrates interaction between this domain and other regions is required for proper PAT function (Montoro et al., 2011). Although, the acyl intermediate happened on the cysteine residue in the DHHC motif study on human DHHC3 showed that mutation of other conserved cysteines in the CRD also decreased its activity (Gottlieb et al., 2015). In addition, cysteine residues within a novel CCX_7−−13_C(S/T) motif downstream of the conserved DHHC-CRD of human PATs DHHC5, DHHC6 and DHHC8 were also proved to be S-acylated (Yang et al., 2010). Therefore, it seems that cysteine residues in the DHHC-CRD as well as other motifs play joint roles in of PATs auto-acylation and subsequent transfer of the fatty acid to their substrate proteins. It is also interesting to note that many residues in the DHHC-CRD domain are reported to determine substrate specificity of a PAT, such as A145 and K148 in Swf1 (Montoro et al., 2011).

Some PATs also have an N-terminal ankyin repeat (AR) domain. Usually two AR containing DHHC PATs are found in per genome, such as in mammals (Fukata et al., 2004), yeast (Roth et al., 2006), fly (Bannan et al., 2008), apicomplexan parasite (Frénal et al., 2013), nematode (Edmonds and Morgan, 2014), and plants (Yuan et al., 2013). It is thought that AR can help these PAT to recognize its specific targets for S-acylation (Lemonidis et al., 2015). However, other functions of AR that are independent from S-acylation were also found in some such PATs (Harada et al., 2003; Hemsley and Grierson, 2011; Yang and Cynader, 2011). For example, AR is essential for Akr1 to interact with Gβγ dimer and to suppress cell cycle arrest induced by β subunit, and this process does not require Akr1 being a functional PAT (Hemsley and Grierson, 2011).

Both the N- and C-termini of all PATs characterized so far are highly variable and cytosolic. The highly variability of N- and C- terminal domains are believed to be essential for substrate specificity of PATs, even though there have no experimental evidence to support this at present (Huang et al., 2009; Greaves et al., 2010; Montoro et al., 2011). Many PATs also have a conserved aspartate-proline-glycine (DPG) motif close to the second TMD, and a threonine-threonine-asparagine-glutamate (TTxE) motif adjacent to the last TMD. Both of them are cytosolic but their roles in the function of PATs are still waiting to be explored. Another important region that contains 16-amino acids and is conserved in 70% eukaryotic PATs is the PaCCT motif (Palmitoyltransferase Conserved C-Terminus). Absence of the PaCCT motif abolished the function of Pfa3 in yeast; the tyrosine residue within this motif of Swf1 is essential for its PAT activity toward Tlg1 (González et al., 2009).

**Figure 2:**
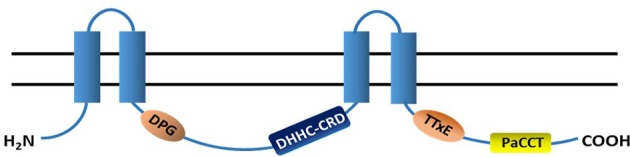
**Topology structure and conserved domains of PATs**. Most PATs have 4 transmembrane domains (TMDs, blue columns) and their N- and C-termini are in the cytoplasm. A highly conserved catalytic DHHC-CRD (aspartate-histidine-histidine-cysteine cysteine rich domain) resides between the 2nd- and 3rd-TMDs. The majority of PATs also have the DPG (aspartate-proline-glycine), TTxE (threonine-threonine-any amino acid-glutamic acid) and PaCCT (Palmitoyltransferase Conserved C-Terminus) domains, and all of them are cytosolic.

The general structure and the functional/conserved domains of PATs is illustrated in Figure 2.

### DHHC proteins are commonly found in eukaryotes

Since the first DHHC containing protein, Akr1 was found and proved to be a PAT from yeast in 2002, significant advances have been made in understanding of DHHC protein family in yeast, mammals, worm and plants. So far, 6 of the 7 yeast DHHC proteins have been confirmed to be PATs, and they are Akr1 (Lobo et al., 2002; Roth et al., 2002), Erf2 (Valdez-Taubas and Pelham, 2005), Swf1 (Smotrys et al., 2005), Pfa3 (Hou et al., 2009), Pfa4 (Smotrys et al., 2005), and Pfa5 (Roth et al., 2006). Akr2 is highly homologous to Akr1 with a typical DHHC-CRD and two ARs, however, *akr2* mutant did not show any remarkable phenotype and there is no direct evidence to show whether it has PAT catalytic activity or not (Kihara et al., 2005; Linder and Deschenes, 2007).

Among the 22 human DHHC proteins (DHHC1-22), 17 were proved to have PAT activities excluding DHHC4, 11, 13, 19, and 22 (Ohno et al., 2012). One more DHHC protein (DHHC23) was found in mice but its homolog cannot be found in human (Ohno et al., 2006; Greaves and Chamberlain, 2010). *Caenorhabditis elegans* has 15 DHHC-PATs, but so far only 1, SPE10 (spermatogenesis 10), was characterized in some detail and showed that it is essential for membranous organelles to deliver fibrous bodies to the spermatid (Gleason et al., 2006).

Plant genomes also possess various numbers of DHHC containing protein sequences. A recent survey from 31 plant species with complete genomes including Arabidopsis, identified 804 DHHC proteins. The numbers of DHHC proteins were variable in different species from 6 in *Volvox carteri* to 52 in *Panicum virgatum*. Expression pattern of DHHC proteins in *Zea mays* and their response to phytohormones and abiotic stress showed that these DHHC proteins may play important roles in plant growth and development as well as stress responses (Yuan et al., 2013). Arabidopsis has 24 DHHC-containing proteins, named as ATPAT1-24 (Hemsley et al., 2005; Batistič, 2012). According to their phylogenetic relationship they are divided into 3 main groups, where group A has the most members including AtPATs 1–9, group B consists AtPATs 11–16, group C is made of AtPATs 18–22, whilst AtPAT10, 17, 23, and 24 do not belong to any groups (Batistič, 2012). All these putative PATs have 4 TMDs except for AtPAT15 and AtPAT17 where 3 and 6 TMDs are found respectively.

Similar to what was found in yeast and mammals, Arabidopsis genome also has 2 ankyrin repeats containing PATs, AtPAT23 and AtPAT24 that are highly homologous. Being the first PAT identified in higher plant, AtPAT24 was confirmed to be an S-acyl transferase because it not only auto-acylated but also rescued the yeast PAT Akr1 knockout mutant *akr1* for its morphological and temperature sensitive defects. AtPAT24 can also restore the correct localization of one of the Akr1 palmitoylating proteins, the yeast casein kinase 2 (Yck2). The AtPAT24 loss-of-function mutant *tip1* exhibites defects in cell size control, pollen tube and root hair growth, as well as cell polarity (Hemsley et al., 2005). Following this, the biological functions of 3 other AtPATs have also been characterized in some detail recently. For example, three T-DNA insertion mutant alleles were identified for AtPAT10 and all showed pleiotropic defects, including cell expansion, cell division, vascular patterning, fertility and salt stress in Arabidopsis (Qi et al., 2013; Zhou et al., 2013). Single mutants of *atpat13* and *atpat14* are semi-dwarf and show precocious leaf senescence, and the double mutant *atpat13 atpat14* has even stronger phenotype than each of their parent single mutant plants (Lai et al., 2015; Li et al., 2016).

DHHC containing proteins were also identified from other organisms. For example, 22 such proteins were found in Drosophila (Bannan et al., 2008), 18 in *T. gondii*, 17 in *Neospora caninum*, 12 in *Trypanosome brucei*, 12 in *P. falciparum*, 11 in *Plasmodium bergbei*, 10 in *Cryptosporidium* species, 9 in *Theileria parva*, 8 in *Babesia bovis* and 6 in *Eimeria tenella* (Frénal et al., 2013). It was reported that TgDHHC7 from *T. gondii* is essential for rhoptry organelles localization and parasite invasion (Frénal et al., 2013).

### Expression pattern and subcellular localization of PATs

The spatial and temporal expression patterns of genes are very important for their cellular functions. However, only very limited information available for the expression patterns of PAT family proteins so far. It was shown that the humans DHHC1, 3–10, 12–14, 16–18, and 20–22, are ubiquitously expressed in different tissues. DHHC19 had very high expression level in testis with weak expression in thymus and small intestine while DHHC11 was only expressed in testis. In addition, only very low level of *DHHC2* transcript was present in kidney and testis, and similar low levels of *DHHC15* transcript were found in heart, brain, lung, kidney, thymus, and small intestine (Ohno et al., 2006).

The expression patterns of DHHC proteins in Drosophila were also analyzed. Among them, CG1407, CG5620, CG6017, CG6627, and CG17257 exhibited maternal expression and were enriched in neural tissues, with transcripts of all of them except for CG1407 also detected in larval brains. Some DHHC proteins were only expressed in testis, such as CG4483, CG4956, CG13029, CG17075, CG17195-17198, CG17287, and CG18810, and others expressed only in ovary, such as CG5880, CG6017, and CG34449 (Bannan et al., 2008).

Study of the expression patterns of PATs in parasite also showed that most of them have ubiquitous expression with a few being more tissue or developmental stage specific. For instance, TgDHHC18 is specially expressed in bradyzoites, TgDHHC10 at oocyst stage, PfDHHC6 and PfDHHC10 at gametocyte stage (López-Barragán et al., 2011; Frénal et al., 2013).

In Arabidopsis, 19 of the 24 *AtPATs* expressed in a broad and constant pattern with transcripts detected in most tissues at all different developmental stages in Arabidopsis. However, *AtPAT1, 2, 3, 11*, and *21* had relatively low expression levels than other *AtPATs*, and *AtPAT2* and *3* also exhibited stronger expression in pollen (Batistič, 2012; Yuan et al., 2013). In *Oryza sative*, 26 of the 30 *OsPATs* can express in more than one type of tissue, among which *OsPAT29* was only expressed during germination stage, *OsPAT21* and *OsPAT26* were only expressed in the internode and stamen respectively. The transcripts of *OsPAT13* and *OsPAT28* were barely detectable in the tissues examined (Yuan et al., 2013). In *Zea mays* 28 of 38 *ZmPATs* have extensive expression in different developmental stages and tissues with *ZmPAT13* and *ZmPAT22* having higher expression in anther (Yuan et al., 2013). However, in *Glycine max*, specific, rather than broad expression patterns were found where 7 *GmPATs* were specifically expressed in the flowering stage and the transcripts of the remaining 11 *GmPATs* were detected in all developmental stages except for flowering stage (Yuan et al., 2013).

Therefore, it is clear that most PATs exhibit a broad expression pattern in different developmental stages and tissues with a few stage or tissue specific PATs in all organisms reported. This suggests that most PATs are involved in a broad range of functions in a given organism, such as Arabidopsis.

PATs distributed in the entire endomembrane system in the cell and this locality nature of PATs may determine the specific set of proteins they modify. For example, the plasma membrane-localized Pfa5 in yeast and DHHC5, 20 and 21 in human are the PATs involved in S-acylation mediated signal transduction of PM localized heterotrimeric G protein alpha subunit Gsα, the β2-adrenergic receptor and endothelial nitric oxide synthase (Mumby et al., 1994; Robinson et al., 1995; Loisel et al., 1996; Ohno et al., 2006). The ER- and Golgi-localized DHHC proteins may be responsible for palmitoylation of de novo synthesized proteins during the processes of membrane localization and delivery to other organelles (Ohno et al., 2006). The tonoplast-localized Pfa3 in yeast palmitoylates Vac8p for its vacuolar membrane targeting (Hou et al., 2005; Smotrys et al., 2005). Therefore, to determine the subcellular localization of individual PAT is very important in order to understand its function by identifying the substrate protein(s) it modifies and signaling pathways it is involved.

Although, PATs are found in all endomembrane systems in the eukaryotic cell they have different preference in different species as to which endomembrane compartment they are localized. For instance, in yeast, 3 PATs, Swf1, Pfa4, and Erf2 are localized at ER; Akr1 and Akr2 are localized at Golgi; while Pfa3 and Pfa5 are localized at vacuole and plasma membrane respectively (Valdez-Taubas and Pelham, 2005; Ohno et al., 2006). In human, 8 PATs are localized at ER (DHHC1, 6, 10, 11, 13, 14, 16, and 19), 7 at Golgi (DHHC3, 4, 7, 8, 15, 17, and 18), 2 at PM (DHHC5 and 20), 4 at both ER and Golgi (DHHC2, 9, 12, and 22), and 1 at both Golgi and PM (DHHC21) (Ohno et al., 2006).

Similar to the mammalian PATs, DHHC-PATs in Drosophila are also mainly localized at ER (14: CG4483, CG4676, CG4956, CG5196, CG5620, CG6627, CG10344, CG13029, CG17075, CG17195, CG17196, CG17197, CG17198, and CG17287) and Golgi (6: CG5880, CG6017, CG6618, CG8314, CG17257, and CG18810). The only exception is CG1407 which is localized at PM (Bannan et al., 2008). In Apicomplexan, such as *T. gondii*, TgDHHCs are not only localized on the common organelles such as Golgi (TgDHHC1, 5, 6, 9, 11, 12, 15, and 17), ER (TgDHHC3, 8, and 16), PM (TgDHHC4 and 13), but also the Apicomplexan-specific organelles such as IMC (TgDHHC2 and 14) and rhoptries (TgDHHC7) (Frénal et al., 2013).

In some contrast to the subcellular localization described above, studies carried out on transiently expressed in tobacco leaves of the 24 Arabidopsis PATs show that 9 of them are localized on PM, including AtPAT04-09, 12, 19, and 21. Therefore, it was proposed that PM is the main site for S-acylation in Arabidopsis plant (Batistič, 2012). This is different from mammalian PATs where most of them are localized in Golgi and therefore Golgi is thought to be the major S-acylation machinery (Ohno et al., 2006; Batistič, 2012). It is also interesting to note that many AtPATs that are localized on ER and PM are also associated with vesicles around them. For example, AtPAT3, 15, 17, and 18 are localized at ER as well as on the vesicles associated with them; AtPAT13, 20, and 22 at PM and also vesicles (Batistič, 2012). AtPAT10, 14, 16, 23, and 24 are mainly localized at Golgi, and the Golgi-localization of AtPAT10 and AtPAT14 were further confirmed in stably transformed Arabidopsis plants (Qi et al., 2013; Li et al., 2016). While the main residence of AtPAT01 and AtPAT02 are the endosomal compartments AtPAT10 and AtPAT11 were found on the tonoplast (Batistič, 2012; Qi et al., 2013; Zhou et al., 2013). It is noteworthy that a few Arabidopsis PATs have dual subcellular localizations, such as AtPAT10 (Golgi and tonoplast) and AtPAT13/20/22 (PM and vesicles) (Batistič, 2012; Qi et al., 2013). Similar observations were also made with some mammalian PATs (Valdez-Taubas and Pelham, 2005; Ohno et al., 2006). However, the significance of this dual-localization nature of PATs is currently unknown.

Little is known about how the PAT proteins achieve their respective localization in the cell. A recent study show that the lysine-based sorting signals KXX and KKXX are present in the mammalian DHHC4 and DHHC6, respectively, and it is these motifs that restrict their localization to the ER (Gorleku et al., 2011). It is also revealed that the C-terminal 68 amino acids of the mammalian DHHC2 play an important role to define its subcellular localization to the ER and Golgi (Fukata et al., 2013). However, there is currently no information available on how plant PAT are targeted to individual membranes in the cell.

### The identified PAT/substrate pairs

As an enzyme PAT carries out its function mainly through substrate protein(s) it S-acylates. Therefore, to understand how PATs operate it is important to identify the target proteins they modify. However, to match an individual PAT and its S-acylated substrate proteins has proved to be a very difficult task so far. This is because: (1) the number of potential S-acylated proteins far exceed the number of their modifying PATs. For example, there are 7 PATs in yeast, however, ~50 S-acylated proteins were identified by a proteomic approach (Roth et al., 2006). Similarly, much more S-acylated proteins were isolated than the number of PATs present in mammals and Arabidopsis (Martin and Cravatt, 2009; Hemsley et al., 2013). Therefore, it seems most likely that at least some if not all PATs can S-acylate multiple substrate proteins, i.e., PATs do not have strict substrate specificity; (2) Many substrate proteins can be modified by more than one PATs. For instance, in yeast, the S-acylation of Vac8 is only partially reduced in the yeast PAT knock-out strain *pfa3*, thus it is most likely that Vac8 is S-acylated by Pfa3 as well as one another or other PATs (Smotrys et al., 2005). Similarly, Ras2 S-acylation is only partially suppressed in the absence of Erf2 hence other PATs are also capable to S-acylate Ras2 (Roth et al., 2006; Montoro et al., 2011). Therefore, these PATs have specific yet overlapping substrate specificity. For some peripheral membrane proteins in mammalian cells, their S-acylation is devoid of specificity altogether (Rocks et al., 2010). However, reports show that some PATs do have their preferentially modified proteins. For example, Swf1 in yeast prefers to function on transmembrane proteins that have cysteines close to TMDs (Roth et al., 2006). In human, integrin α4β6 is strictly modified by DHHC3 (Sharma et al., 2012); (3) No consensus sequences in S-acylated proteins have been found. Although many S-acylated proteins have been identified and some of them are S-acylated by the same PAT, there are no consensus sequences characterized in these proteins (Montoro et al., 2011).

In yeast, each of the five PATs have been mapped to one or more substrate proteins. However, the total number of these individual substrate proteins are still far less than ~50 S-acylated proteins identified (Roth et al., 2006). For example, Akr1 S-acylates casein kinases Yck1, Yck2, and Yck3 (Roth et al., 2002). It also S-acylates sphingosine kinase Lcb4 because 60-80% of reduction in S-acylation of Lcb4 was found in *akr1* mutant yeast (Kihara et al., 2005). Other proteins that are also S-acylated by Akr1 are Meh1, Sna4 and the unknown function proteins such as Ypl199c, Ykl047w, and Ypl236c (Roth et al., 2006). Therefore, Akr1 alone can S-acylate at least 9 substrate proteins in yeast. It was noted that Akr1 prefers hydrophilic proteins that tether to membranes solely through N- or C-terminal palmitoyl modifications (Roth et al., 2006). Erf2 is responsible for the S-acylation of Ras and other signaling proteins such as Rho2, Rho3, Gpa1, Gpa2, and Ste18, all of which are heterolipidated (Bartels et al., 1999; Lobo et al., 2002; Roth et al., 2006; Zhang et al., 2013). Swf1 tends to S-acylate proteins that have juxta-TMD mapping cysteines, such as SNAREs (Valdez-Taubas and Pelham, 2005), mannosyltransferases including Mnn1, Mnn10 and Mnn11 and prion induction protein Pin2 (Roth et al., 2006). Pfa4 is devoted to the palmitoylation of a group of Amino Acid Permeases (AAPs). AAPs is a family of plasma membrane transporters with 12 TMDs and a conserved C-terminal Phe-Trp-Cys palmitoylation site. Experiments in *C. neoformans* showed that Pfa4 is also responsible for PM localization of Ras1 via palmitoylation (Merino et al., 2014). On the other hand, one substrate protein can be palmitoylated by multiple PATs. For example, S-acylation of Gpa2 is mediated by both Pfa5 and Erf2 (Roth et al., 2006; Greaves and Chamberlain, 2011; Zhang et al., 2013); Meh1 was S-acylated by Pfa3 and Akr1 (Greaves and Chamberlain, 2011); and an unknown protein Yg1108 was S-acylated equally by Erf2 and Pfa4 (Greaves and Chamberlain, 2011). However, so far the substrates of Akr2 has not been identified. Therefore, it is clear that both PATs and their substrate proteins are highly redundant in yeast. A summary of PATs and their substrate proteins in yeast is shown in Table 4.

**Table 4:** **Substrates of yeast PATs**.

**PATs**	**Substrates**	**References**
Akr1	Lcb4, Yck1, Yck2, Yck3, Meh1, Sna4, Ypl199c, Ykl047w, Ypl236c, Vac8	Roth et al., 2002; Babu et al., 2004; Kihara et al., 2005; Roth et al., 2006, 2011
Erf2 (shr5)	Ras1, Ras2, Rho2, Rho3, Gpa1, Gpa2, Ste18, Ycp4, Psr1, Yg1108	Bartels et al., 1999; Lobo et al., 2002; Ohno et al., 2006; Roth et al., 2006; Greaves and Chamberlain, 2011; Zhang et al., 2013
Swf1	Many SNAREs, Mnn1, Mnn10, Mnn11, Pin2	(Valdez-Taubas and Pelham, 2005; Roth et al., 2006)
Pfa3	Vac8, Meh1	Hou et al., 2005; Smotrys et al., 2005; Roth et al., 2006
Pfa4	APPs, Lcb4, Ras1,Yg1108	Ohno et al., 2006; Roth et al., 2006; Greaves and Chamberlain, 2011; Nichols et al., 2015
Pfa5	Gpa2	Greaves and Chamberlain, 2011

Many substrate proteins of mammalian PATs have also been identified in recent years. These include GTP-binding proteins, cytoskeletal proteins, enzymes, neurotransmitter receptors and synaptic scaffolding proteins (Table 5). Similar to what is found in yeast, some proteins can be modified by more than one PAT and most PATs can modify multiple proteins such as DHHC2, 3, 5, 7, 8, 13, 15, 17, and 21 (Table 5). For instance, PSD-95, a protein that scaffolds receptors and signaling enzymes at the postsynapse (Topinka and Bredt, 1998) can be S-acylated by DHHC2, 3, 7, 8, 15, and 17 (Fukata et al., 2004, 2006; Greaves and Chamberlain, 2011; Butland et al., 2014). SNAP-25, a t-SNARE protein that regulates neurotransmitter release, is the substrate of DHHC2, 3, 7, 8, 15, and 17 (Greaves et al., 2009). S-acylation of a tyrosine kinase Fyn is mediated by DHHC2, 3, 7, 15, 20, and 21 (Mill et al., 2009). All these mentioned PAT/substrates and other pairs are listed in Table 5. On the other hand, one PAT can palmitoylate multiple substrate proteins. For example, DHHC2 palmitoylats cytoskeleton-associated protein 4 (CKAP4) and AKAP79/150 (Keith et al., 2012; Chavda et al., 2014); DHHC3 does integrin α6β4, Calmodulin-dependent protein kinase isoform 1γ (CaMKIγ), NMDA receptor subunits 2A and 2B (NR2A/B) and DR4 Takemoto-Kimura et al., 2007; Hayashi et al., 2009; Sharma et al., 2012; Yeste-Velasco et al., 2015; the S-acylation of Grip1b, δ-catenin, Flotillin-2, somatostatin receptor 5 and Ankyrin-G is carried out by DHHC5 (Brigidi et al., 2015). In the same fashion many other proteins are also palmitoylated by specific PATs (Table 5). Importantly, some DHHC proteins have been indicated to be involved in certain diseases, such as DHHC8 in schizophrenia, DHHC9 and 15 in X-linked mental retardation, DHHC17 in Huntington's disease and many PATs are involved in different types of cancer including DHHC2, 3, 7, 9, 11, 14, 17, 20, and 21 (Chavda et al., 2014; Yeste-Velasco et al., 2015). However, for some of them, their specific substrate proteins have not been identified.

**Table 5:** **Mammalian PATs and their (regulated) target proteins**.

**PAT**	**Targets**	**References**
DHHC2	PSD-95, CKAP4, SNAP23/25, eNOS, Fyn, NDE1, NDEL1, CD9/151, ABCA1, AKAP79/150	Fukata et al., 2004; Huang et al., 2004; Fernández-Hernando et al., 2006; Sharma et al., 2008; Zhang et al., 2008; Shmueli et al., 2010; Singaraja et al., 2009; Greaves et al., 2010; Chavda et al., 2014
DHHC3 (GODZ)	PSD-95, SNAP23/25/25b, Gα, CSP, Integrin α6β4, GABA_*A*_γ2, eNOS, GluR1/2, GAD65, STREX, Fyn, BACE1, NDE1, NDEL1, NCAM140, CaMKIγ, NR2A/B, DR4, PI4KII	Fukata et al., 2004; Keller et al., 2004; Hayashi et al., 2005; Fang et al., 2006; Fernández-Hernando et al., 2006; Takemoto-Kimura et al., 2007; Greaves et al., 2008; Huang et al., 2009; Mill et al., 2009; Tsutsumi et al., 2009; Vetrivel et al., 2009; Greaves et al., 2010; Tian et al., 2010; Shmueli et al., 2010; Yeste-Velasco et al., 2015
DHHC4	BACE1	Vetrivel et al., 2009
DHHC5	Grip1b, δ-catenin, Flotillin-2, somatostatin receptor 5, Ankyrin-G, STREX	Tian et al., 2010; Kokkola et al., 2011; Li et al., 2012; Thomas et al., 2012; Brigidi et al., 2014, 2015
DHHC6	Chaperone calnexin	Lakkaraju et al., 2012
DHHC7	PSD-95, Gα, CSP, Fyn, eNOS, SNAP25/23/25b, GABAAγ2, STREX, BACE1, NDE1, NDEL1, NCAM140, sortillin, PDE10A2, CD9, ER, PR, AR, PI4KII	Fukata et al., 2004; Fang et al., 2006; Fernández-Hernando et al., 2006; Fukata et al., 2006; Greaves et al., 2008; Mccormick et al., 2008; Ponimaskin et al., 2008; Greaves et al., 2009; Tsutsumi et al., 2009; Vetrivel et al., 2009; Shmueli et al., 2010; Charych et al., 2010; Greaves et al., 2010; Tian et al., 2010; Ohno et al., 2012
DHHC8	eNOS, SNAP25, paralemmin-1, GAD65, PSD95, PSD93	Fernández-Hernando et al., 2006; Mukai et al., 2008; Huang et al., 2009
DHHC9	H- and N-Ras, STREX	Swarthout et al., 2005; Tian et al., 2010
DHHC12	ABCA1	Singaraja et al., 2009; Chavda et al., 2014
DHHC13 (HIP14L)	MT1-MMP, HTT, GAD65	Huang et al., 2009; Saleem et al., 2010; Song et al., 2014
DHHC15	PSD95, GAP43, SNAP25b, CSP, GABA_*A*_γ2, Fyn, BACE1, CD151, CI-MPR, sortillin	Fukata et al., 2004; Fang et al., 2006; Greaves et al., 2008; Mccormick et al., 2008; Sharma et al., 2008; Mill et al., 2009; Vetrivel et al., 2009; Greaves et al., 2010
DHHC17 (HIP14)	PSD95, CLIP3, CSP, GAD65, GAP43, GLUR1/2, GPM6A, HTT, JNK3, Lck, SNAP25/23/25b, STREX, SYT1, SPRED1/3, Ras	Fukata et al., 2004; Ohyama et al., 2007; Greaves et al., 2008; Huang et al., 2009; Mill et al., 2009; Greaves et al., 2010; Tian et al., 2010; Ohno et al., 2012; Butland et al., 2014
DHHC18	H- and N-Ras, Lck	Fukata et al., 2004
DHHC19	R-Ras, PDE10A2	Baumgart et al., 2010; Charych et al., 2010
DHHC20	Fyn, BACE1, ABCA1	Mill et al., 2009; Singaraja et al., 2009; Vetrivel et al., 2009
DHHC21	PECAM1, SOD1, Lck, eNOS, Fyn, ABCA1, ER, PR, AR	Fernández-Hernando et al., 2006; Takemoto-Kimura et al., 2007; Mill et al., 2009; Vetrivel et al., 2009; Antinone et al., 2013; Akimzhanov and Boehning, 2015; Yeste-Velasco et al., 2015

Very little information is available for PAT/substrate pairs in other organisms. The only PAT/substrate pair characterized was in *P. falciparum* where PfDHHC1, an apicomplexan-specific and inner membrane complex-localized PAT, has identical expression pattern to two S-acylated proteins PfISP1 and PfISP3 (Wetzel et al., 2015).

In plant, the only putative PAT/substrates pairing identified is ATPAT10/AtCBL2, 3, 6. This was achieved by transient expression of AtCBL2, AtCBL3 and AtCBL6 in Arabidopsis protoplast, showing that the tonoplast localization of AtCBLs is lost in protoplast prepared from AtPAT10 loss-of-function mutant (Zhou et al., 2013).

Therefore, although many hundreds of S-acylated proteins, including putative ones isolated by large proteomic approaches were identified from different species at present, there are many more to come in the future due to the readily available proteomics facilities in large institutions. A framework for characterizing PAT/substrate selectivity is urgently required to set out to match individual PATs and their S-acylated substrate proteins in order to understand the mechanism of S-acylation in individual organism and in general.

## De-S-acylation

Similar to phosphorylation and ubiquitiation, S-acylation process is reversible, which makes it a very important lipid modification of proteins (Hemsley, 2009). S-acylation turnover by de-S-acylation, can be constitutive or stimulated (Smotrys and Linder, 2004). Ras proteins were the first proteins to be reported to have dynamic S-acylation with different H-Ras has different de-S-acylation rates (Baker et al., 2003). S-acylation/ de-S-acylation of Fyn, a member of the Src kinase family, happens with a half-life of 1.5–2 h (Wolven et al., 1997; Zeidman et al., 2009). The de-S-acylation of Gα subunits is stimulated by the activation of G-protein-coupled receptors (Mumby et al., 1994; Linder and Deschenes, 2007). De-S-acylation of PSD-95 is enhanced by neuronal activity (El-Husseini et al., 2002).

At present only four protein thioesterases have been identified to catalyze the de-S-acylation process, including acyl protein thioesterases 1 (APT1) and 2 (APT2), palmitoyl thioesterases 1 (PPT1) and 2 (PPT2) (Tomatis et al., 2010; Hornemann, 2015). These enzymes carry out the de-S-acylation step in which the palmitate or other long chain fatty acids are removed from the S-acylated proteins (Linder and Deschenes, 2007). APT1 was first found in rat liver as a lysophospholipase and its substrates include Ras, Gα subunit, RGS4, SNAP-23, and eNOS (Toyoda et al., 1999; Akimzhanov and Boehning, 2015). APT2 was reported to de-S-acylate the growth-associated protein 43 (Tomatis et al., 2010). PPT1 is a soluble lipase that is localized in lysosomes and it is responsible for the degradation of S-acylated proteins (Linder and Deschenes, 2007; Chavda et al., 2014). The loss-of-function of PPT1 resulted in severe infantile neuronal ceroid lipofuscinosis (Vesa et al., 1995). PPT2 has very limited acyl protein thioesterase activity, which prefers de-S-acylating short-chain lipid substrate. Interestingly, study has shown that it is up-regulated in obesity (Bürger et al., 2012; Fox et al., 2012).

It is surprising that only four thioesterases have been identified so far yet many hundreds of S-acylated proteins were isolated from different genomes. The explanations for this could be: (1) thioesterases are broad specificity enzymes, each of which can de-S-acylate a wide range of substrates; (2) not all S-acylated proteins undergo de-S-acylation; (3) of course, it could be because many more thioesterases have not been found (Chavda et al., 2014). There currently no protein thioesterases have been identified from plant.

## Mechanism of protein S-acylation

It is well recognized that DHHC proteins transfer acyl group via a two-step catalytic mechanism in which the enzyme first modifies itself with palmitate (or other long chain fatty acids) in a process termed autoacylation. The enzyme then transfers the acyl group from itself onto its substrate proteins. However, the number and location of the S-acylated cysteines of a given PAT in the autoacylated intermediate is unknown. It is well accepted that the cysteine in the DHHC motif is the auto-S-acylation site because mutation in this residue results in loss of auto-acylation of many characterized PATs from yeast (Montoro et al., 2011), mammals (Ohno et al., 2012), and Arabidopsis (Hemsley et al., 2005; Qi et al., 2013). However, cysteines in other positions of PATs such as the CRD and other domains may also be autoacylated (Gottlieb et al., 2015). For instance, DHHC3 has 6 auto-S-acylation sites where 5 in the CRD, including Cys-132, Cys-133, Cys-146, Cys-157, and Cys-163, 1 in the N-terminal domain (Cys-24) (Gottlieb et al., 2015).

### Techniques used for prediction and confirmation of S-acylated cysteines in proteins

There is no consensus for sequences in the S-acylated proteins despite that many such proteins have been isolated through proteomics approach or individually confirmed via radioactive labeling or/and mutation studies. Nevertheless, it is noted that: (1) in some S-acylated soluble proteins the cysteine residues that are S-acylated are frequently surrounded by basic or hydrophobic amino acids, such as GAP-43 (Liu et al., 1993) at N-terminal motif, Yck2 (Roth et al., 2002) at C-terminal motif and SNAP-25b (Lane and Liu, 1997) at cysteine string motif (Smotrys and Linder, 2004); (2) in other S-acylated soluble proteins the Cys residue is located near the prenylated or myristoylated residues, resulting in the so-called dual lipidition. These proteins include the Arabidopsis α and γ subunits of heterotrimeric G protein (Adjobo-Hermans et al., 2006; Zeng et al., 2007), small GTPases (Deschenes et al., 1990; Bartels et al., 1999; Roth et al., 2006; Zhang et al., 2013); 3 for transmembrane proteins, the Cys residues are often situated in the cytoplasmic regions of membrane-spanning regions (Roth et al., 2006; Ohno et al., 2012). For instance, the S-acylation of C261-263 triplet in death receptor 4 (DR4) (Rossin et al., 2009) and C474 in β-secretase BACE1 (Motoki et al., 2012) promotes their association with lipid raft.

Based on the above information several software packages have been developed to predict the S-acylated cysteines, such as a clustering and scoring strategy known as CSS-Palm (Zhou et al., 2006), which has been updated to the latest version CSS-Palm 4.0 (freely available at http://csspalm.biocuckoo.org/), incremental feature selection (IFS)-Palm (Hu et al., 2011), weight, amino acid composition and position specific scoring (WAP)-Palm (Shi et al., 2013) and PalmPred (Kumari et al., 2014). All of them are on-line so that one can input the protein sequence of interest to predict the possibility of its S-acylation and where the Cys residues are located within the sequence. The prediction data from these platforms can then be confirmed experimentally. These techniques include:

1. PAT inhibitors. The palmitate analog, 2-bromopalmitate (2-BP) is the most commonly used inhibitor of S-acylation, which inhibits palmitoylation in cells and PAT activity of DHHC proteins *in vitro* (Webb et al., 2000; Fukata et al., 2004; Jennings et al., 2009). However, it lacks specificity and can also inhibit myristoylation and reduce de-acylation through inhibiting activities of acyl-protein thioesterases (Webb et al., 2000; Pedro et al., 2013). Tunicamycin and cerulenin are also used to inhibit S-acylation, but similar effect was found as 2-BP (Patterson and Skene, 1995; Lawrence et al., 1999). Recently, a compound, 2-(2-hydroxy-5-nitro-benzylidene)-benzo[*b*]thiophen-3-one, was shown to have more specificity, but it does not have selectivity for specific PAT, i.e., it inhibits activities of all PATs (Jennings et al., 2009), which means it still cannot be used to study the function of individual PAT. Therefore, it is clear that results obtained from these inhibitors should be further validated by mutational or biochemical analysis.
2. Mutational analysis to change the potential S-acylated Cysteine to alanine or serine. Both alanine and serine were frequently used to replace the cysteine to achieve similar results (Hemsley et al., 2005; Qi et al., 2013; Li et al., 2016). Cysteine and serine have very similar structure, when the cysteine is mutated to serine it can maintain the size and the properties of the putative S-acylated protein, in this case, serine is a better substitution for cysteine. However, compare to alanine, serine is more hydrophilic than cysteine and might also cause unwanted side chain effects (Nagano et al., 1999). In this specific study, both alanine and serine as the substitutions for cysteine are accepted so far. This is followed by comparing the effect on the differences of functions or the subcellular localizations to native protein. If a difference was found the proteins were most likely S-acylated at the cysteine residues that were mutated.
3. Biochemical assays to analyze the attachment of fatty acids of the individual proteins. This includes: (1) traditionally feed with tritiated fatty acids followed by exposures to X-ray film (Lavy et al., 2002); (2) azido-alkyne CLICK-chemistry (Martin and Cravatt, 2009); (3) Acyl-exchange, or Biotin-switch assay (Wan et al., 2007; Hemsley et al., 2008); and (4) direct resin immobilization (Forrester et al., 2011).
4. Direct detection of the S-acyl group by gas chromatography–mass spectrometry (GC-MS) analysis. The identification of lipid groups attached to proteins can help to understand the biophysical properties of the protein. This method has been successfully used to demonstrate S-acylation of CBL1 and CBL2, which are attached by palmitate and/or stearate (Batistic et al., 2008, 2012).

### Specificities of PAT-substrate interaction

Although, it is generally accepted that PATs are lacking specificity toward their substrate proteins and vice versa studies in yeast showed that some PATs do exhibit preference to some substrate protein(s) compared than others. For instance, Akr1 prefers to S-acylate soluble proteins at their N- or C-terminus, such as Ypl236c S-acylated at N-terminal cysteine and Yck1 S-acylated at C-terminal cysteine. Erf2 and Pfa5 show preference for pre-lipidated substrates, such as prenylated Ras1 and Ras2, myristoylated Gpa1 and Gpa2. Swf1 and Pfa4, on the other hand, prefer single and multiple transmembrane proteins such as SNAREs and AAPs (Roth et al., 2006; Ohno et al., 2012). Similar conclusions were made from studies of mammalian DHHC proteins where it was found that DHHC3, 7, 8, and 14–17 had high activities toward soluble proteins, while DHHC2, 20 and 21 were highly active to integral membrane proteins (Ohno et al., 2012). However, it was also noted that most DHHC proteins in both yeast and mammals had overlap activity to modify pre-lipidated substrates, such as 4 yeast PATs and 16 mammalian PATs can all S-acylate the myristoylated Gpa2 (Ohno et al., 2012).

The question here is how PATs and their substrates recognize each other? To address this, studies were carried out on some PATs and their S-acylated proteins in mammalian system. It was reported that the AR domain of the two ankyrin repeat containing PATs DHHC13 and 17 in mammals can act as substrate-recruiting signal and recognizes the [VIAP][VIT]XXQP motif that is shared between some S-acylated proteins including SNAP25/23, CSP, HTT, and CLIP3 (Lemonidis et al., 2015). Fusing the AR domain of DHHC17 to the N terminus of DHHC3 that lacks an AR domain, can make DHHC3 a PAT for HTT which also supports the notion that the AR domain contributes to the substrate specificity of DHHC17 for HTT (Huang et al., 2009). DHHC3 and 7 interact with GABA_*A*_γ2 through a 14-amino acid cysteine rich domain (Fang et al., 2006). DHHC7 has two splicing isoforms, the longer one has additional 111 bp compared to the shorter one, which might possess its tissue-specific function since it expresses specifically in placenta, lung, liver, thymus and small intestine (Ohno et al., 2006). The recognition and S-acylation of PSD95 by DHHC17 depend on the N-terminal 13 amino acids of PSD-95 (Huang et al., 2009). The cysteine rich “CCPCC” motif of PI4KII is required for its S-acylation by DHHC3 and DHHC7 (Lu et al., 2012). Subtle changes in the S-acylation domains of proteins can alter their PAT specificity, which were proved from SNAP23/25 (Greaves et al., 2010). For instance, a SNAP25 mutant which lacks a proline located 25 residues downstream of the S-acylated domain can only be modified by DHHC3 but not DHHC17 (Greaves et al., 2009). Therefore, specific domains in PATs and their substrate proteins are required for recognition and S-acylation to occur.

Subcellular localizations of PATs can have a profound effect on the type of proteins it can S-acylate (Greaves and Chamberlain, 2011). A transmembrane protein might only have access to be S-acylated by the PATs localized on the same membrane. For example, the PM-localized Gpa1 is S-acylated by the PM-localized Pfa5 in yeast (Ohno et al., 2006); tonoplast-localization of AtCBL2 and AtCBL3 is via S-acylation carried out by AtPAT10 which is also localized on tonoplast in Arabidopsis (Zhou et al., 2013).

For S-acylation of a protein to occur its prior membrane attachment via TMD, another lipid modification or protein-protein interaction is often acquired (Hemsley, 2015). For instance, TEM8 localizes at PM with one TMD, S-acylation of which negatively regulate its raft association (Abrami et al., 2006). Some proteins require another lipid modification such as myristoylation to target to certain membrane first before the S-acylation can take place. AtCBL1 is one of these proteins where myristoylation targets it on the ER, then the unknown ER-localized PAT S-acylates the myristoylated AtCBL1. This dual-lipidated AtCBL1 can subsequently be trafficked to the PM (Batistic et al., 2008). It was reported that the N-terminal 12-amino acid peptide of AtCBLs is sufficient to mediate the dual lipid modification and target to PM or tonoplast (Batistic et al., 2008). Therefore, the localization of a specific PAT for a given S-acylated substrate protein relies on where this protein is localized after the first lipid modification.

### Important molecules that are involved in S-acylation

Special molecules have either positive or negative effect on S-acylation of certain proteins. These molecules could be another protein, hormone, ions or protein inhibitors. For instance, although most DHHC-PATs in mammals can catalyze S-acylation independently DHHC9 needs a Golgi-localized protein GCP16 to specially palmitoylate H- and N-Ras (Swarthout et al., 2005). Ykt6, which possibly works as a co-factor of Pfa3 enhanced the S-acylation and vacuole localization of Vac8 (Dietrich et al., 2004; Hou et al., 2005; Meiringer and Ungermann, 2006). S-acylation and localization of Ras protein is catalyzed by the Erf2p-Erf4p complex in yeast (Zhao et al., 2002). Zinc ion is tightly bound to the cysteine rich domain of the DHHC3, which is essential for its structural integrity and PAT activity (Gottlieb et al., 2015). Selenoprotein K (SelK), an 11-kDa endoplasmic reticulum protein of unknown function (Shchedrina et al., 2011) is required for the S-acylation of both IP3R (inositol-1, 4, 5-triphosphate receptor) and CD36 (Fredericks and Hoffmann, 2015). S-acylation of PI4KII is cholesterol-dependent (Lu et al., 2012). Some compounds might have negative effect to specific S-acylation, such as curcumin can prevent S-acylation of integrin β4 by DHHC3 in breast cancer cells (Coleman et al., 2015). Understanding the involvement of these molecules in S-acylation could provide important information in designing and developing new drugs to target the disease and cancer-related S-acylation machinery.

## Conclusion and future perspectives

Ever since the discovery of the first S-acyltransferase, Akr1 from yeast in 2002, which lead to the realization of protein S-acylation being an enzymatic process rather than a simultaneous addition of a long chain fatty acid to proteins, research on S-acylation of proteins has accelerated in a remarkable speed in the past decade. Yeast, as a simple unicellular model eukaryote, has been the first choice for researchers to study S-acylation. The knowledge learnt from the yeast system has then been applied in guiding similar studies in other organisms. As such the important roles of protein S-acylation in growth and development, especially in different human diseases, such as cancers, have hence attracted much attention and become a hot area of research in recent years.

Although, progress has been made toward understanding various aspects of protein palmitoylation the corresponding research in plants is trailing behind that in yeast and mammals. Judging from the wide arrays of S-acylated proteins identified recently from Arabidopsis and Poplar by proteomic studies it is clear that S-acylation plays variable and important roles in plant growth, development and environmental adaption (Hemsley et al., 2013; Srivastava et al., 2016). The knowledge learnt and methodologies developed from yeast and mammals will no doubt provide important clues and necessary tools for us to conduct more efficient research on S-acylation in plants in the coming years. Specifically, (1) the roles of the remaining 21 AtPATs in Arabidopsis will need to be characterized. PATs from other plant species, such as poplar, especially with its S-acylated proteins being isolated recently, will also need to be characterized to see if plant PATs share functional similarity, this will further validate the data obtained from Arabidopsis PATs so far; (2) To match individual PATs with their S-acylated substrate proteins in Arabidopsis and poplar. At present, the only plant PAT with tentative mapped substrate proteins is the Arabidopsis AtPAT10 where it was found that the tonoplast localization of transiently expressed CBL2,3,6 were lost in the protoplast prepared from leaf cells of AtPAT10 loss-of-function mutant plant (Zhou et al., 2013). This indicates that AtPAT10 functions in calcium signaling and salt stress through the actions of these CBLs. Similar approaches could be used to map other PAT/substrate(s) pairs in Arabidopsis and poplar. This will provide further insights to substrate specificity of PATs and molecular mechanisms how PATs function in plants; (3) De-palmitoylation enzymes. S-acylation of proteins is a reversible process where S-acylation is catalyzed by PATs and De-palmitoylation is by acyl protein thioesterases. While 4 such enzymes have been identified and characterized from mammals none from plant. Therefore, research in this area is paramount.

## Author contributions

YL and BQ both contributed in the writing and approving it for publication.

## Funding

This review was produced as part of project about “Protein S-acyl transferases,” which was funded by National Natural Science Foundation of China (Grant No. 31170233 to BQ).

### Conflict of interest statement

The authors declare that the research was conducted in the absence of any commercial or financial relationships that could be construed as a potential conflict of interest.
